# Shelter to Survival: Unpacking the Health Impacts of Housing Insecurity Across the Life Course

**DOI:** 10.3390/ijerph23010091

**Published:** 2026-01-09

**Authors:** Gordane V. Calloo, Mavis Odei Boateng, Eyram A. Agbe, Godfred O. Boateng

**Affiliations:** 1 School of Global Health, York University, North York, ON M3J 1P3, Canada; gordanec@my.yorku.ca; 2 School of Social Work, University of Windsor, Windsor, ON N9A 0C5, Canada; odeiboam@uwindsor.ca; 3 Department of Geography and Environmental Studies, Carleton University, Ottawa, ON K1S 5B6, Canada; eyramagbe@cmail.carleton.ca; 4 Dahdaleh Institute for Global Health Research, York University, North York, ON M3J 1P3, Canada

**Keywords:** housing insecurity, health outcomes, life course, health disparities, rapid review

## Abstract

Housing insecurity is a growing public health concern linked to adverse health outcomes and lifelong vulnerability. Although housing is a well-established social determinant of health, this review employs a life-course framework to explain how housing insecurity contributes to the accumulation of health inequities and chronic disparities across the different stages of human development. A rapid review was conducted across PubMed, Google Scholar, SCOPUS, and Web of Science, focusing on peer-reviewed studies published between 1991 and 2025. Studies were screened using predefined eligibility criteria, and the selection process was documented through a PRISMA flow diagram. Fifty-five studies met the inclusion criteria. Housing insecurity was consistently associated with adverse health outcomes across pregnancy, infancy, childhood, adolescence, adulthood, and older age. Each life stage presents distinct vulnerabilities shaped by environmental and social conditions, which are manifested through physiological and psychosocial pathways. While physical health effects were most frequently reported, developmental and mental health impacts accumulated over time, amplifying overall burden. The findings demonstrate a clear life-course pathway linking housing insecurity to immediate and long-term health risks. Early-life disadvantages create lasting, preventable consequences, underscoring the urgent need for policies that embed housing stability within broader public health planning.

## 1. Introduction

Housing insecurity—characterized by unaffordable housing costs, overcrowding, poor housing quality, and residential instability—is recognized as a critical social determinant of health with profound implications for physical, mental, and developmental outcomes across the lifespan [1]. As Swope and Hernández highlight, four key dimensions of housing insecurity—cost (housing affordability), conditions (housing quality), consistency (residential stability), and context (neighbourhood opportunity)—are essential to understanding this issue [2]. These dimensions interact with structural inequalities, leading to limited or uncertain access to stable, safe, adequate, and affordable housing [3]. As a result, housing insecurity functions not only as material deprivation but as a socially patterned exposure that reinforces existing inequities in access to health-promoting environments. Housing insecurity is a key mediating pathway through which upstream structural conditions are expressed as downstream health inequities [1].

Recent global and national disruptions have intensified housing insecurity’s prevalence and its health consequences, operating through pathways that connect large-scale events to localized housing impacts. Climate change and the recent COVID-19 pandemic have worsened health outcomes primarily by destabilizing housing conditions and intensifying insecurity. This emerges as displacement, overcrowding, and quality degradation, which then generate biological and social vulnerability. Climate change has emerged as a major upstream driver, particularly through climate-related disasters such as flooding, which disproportionately affects vulnerable communities and compromises housing quality, safety and stability [4,5]. Flood-related displacement has been linked to a wide range of adverse outcomes, including mental health disorders, communicable diseases and various non-communicable conditions [4]. Evidence from informal settlements shows especially severe effects: in Indonesia, flooding has produced long-term physical and psychological consequences, including a 78% increase in emotional difficulties among children [5], while across the Global South, structurally weak housing in flood-prone areas has transformed everyday living into hazardous conditions [6]. Similar patterns are evident in high-income countries; in the United States, severe flooding has been associated with increased hospitalizations for injuries, infections, and mental health conditions, underscoring how environmental pressures undermine housing safety and population health [7]. Concurrently, the COVID-19 pandemic further intensified these dynamics by amplifying housing instability and affordability crises, particularly in places such as Canada, where rents and home prices surged, disproportionately burdening low-income households [8]. Collectively, these macro-level disruptions illustrate how global and national forces produce housing insecurity through which health risks are unevenly disrupted.

Housing insecurity operates through determinants spanning multiple ecological levels, requiring examination of how macro-level forces shape local housing conditions and individual exposures. At the structural level, historical policies including redlining, discriminatory lending practices, and exclusionary zoning have systematically concentrated housing instability among racialized and economically marginalized populations [2,9,10]. These legacies persist through contemporary mechanisms: residential segregation continues to limit housing options for racial minorities, while gentrification displaces long-term residents from appreciating neighbourhoods [2,11]. At the neighbourhood or community level, concentrated poverty and disinvestment create environments where affordable housing options are simultaneously scarce and of poor quality [11], exposing residents to environmental hazards including inadequate ventilation, mould, and lead paint [12,13,14,15,16,17]. At the household and individual levels, these structural and neighbourhood conditions manifest as unaffordable rent burdens, overcrowding, frequent moves, and homelessness—proximal housing insecurity exposures that directly impact health through material deprivation, chronic stress, and environmental toxins [1,2,3,16,17]. Understanding these multilevel determinants illuminates how upstream structural conditions produce downstream health inequities, revealing housing insecurity not as an isolated individual problem but as a systematic exposure reflecting broader structural forces. Here, housing insecurity is established as a mechanism through which structural forces become embodied health risk factors and addressing these disparities necessitates targeted policies that recognize housing insecurity as a fundamental determinant of health. Taken together, housing insecurity reflects broader systemic inequities which shape developmental and health trajectories [18,19]. These conditions generate cumulative psychosocial and physiological stress, contributing to biological embedding over time [20]. Chronic exposure to housing-related stressors has been linked to increased allostatic load—the physiological “wear-and-tear” from prolonged stress—which disrupts immune, cardiovascular and metabolic functioning [20,21].

The health consequences of housing insecurity are evident, with timing, duration, and accumulation of exposure playing critical roles. During the perinatal period and early childhood, the causal relationships between the environmental housing defects and poor health outcomes have been associated with developmental delays, lead exposure and increased susceptibility to chronic conditions linked to poor housing quality [15,16,17]. Persistent housing insecurity from infancy through adolescence has been linked to increased mental health issues and chronic diseases, emphasizing the long-term consequences of unstable living conditions [22]. Among adults, unaffordable housing and substandard conditions have been associated with severe maternal morbidity, respiratory illness and compounding physical and psychological burdens [23,24].

Despite growing evidence documenting these associations, much of the existing literature examines housing-related health effects in isolation, without situating them within a life-course continuum. Thus, addressing housing insecurity as a dynamic and cumulative social determinant of health across the lifespan is essential for understanding how health vulnerabilities emerge, persist, and manifest at different life stages.

### Theoretical Framework: Trajectory and Transition

A life-course framework considers the accumulated effects of experiences across the lifespan in determining health maintenance and the onset of disease [25]. This perspective proposes that socially patterned environmental exposures influence the development of biological, physiological, and psychosocial systems, warranting further integration into research on how biological mechanisms contribute to health disparities. The key component of the life course framework understands that the temporality across all stages of a person’s life is intricately intertwined during critical periods at each level of life development [25,26]. Life course theory considers the attribution of housing insecurity as a causative factor of comorbidities at different critical periods over a life span [25,26]. The empirical and conceptual bridge observed between social positionality and the converging relationship of human health creates a gap in health equity. Each developmental stage is sensitive to both biological and social embedding of environmental exposures [26]. Conceptualizing these developmental stages as critical periods provides insight into how risk factors influence health trajectories across the lifespan [27]. This highlights how differential exposure to material and social conditions—shaped by socioeconomic position, racialization, and place—becomes biologically and socially embedded [26,27]. Housing insecurity is well-suited for analysis through a life course lens because it represents a recurrent, structurally patterned exposure that often spans multiple life stages. Experiences of unaffordable housing, poor housing quality, overcrowding and residential instability reflect the broader social stratification by socioeconomic status, race and geographic regions [28,29]. From a life-course perspective, housing insecurity can alter biological and behavioural trajectories by shaping early-life development and constraining adaptive processes, thereby increasing vulnerability to adverse health outcomes later in life [30,31]. The concept of critical periods provides a lens for understanding how exposure to housing insecurity during sensitive developmental windows may have disproportionate effects, while the accumulation of repeated or prolonged exposure across life stages further amplifies health risk [27]. These processes contribute to the reproduction of health inequities across generations, linking individual life trajectories to broader social and policy contexts [26,32,33,34,35].

This framework aligns with global policy priorities articulated in the United Nations Sustainable Development Goals, particularly Goal 3 (good health and well-being) and Goal 11 (sustainable cities and communities), which emphasize access to adequate, safe, and affordable housing as foundational to population health [36]. Together, the adoption of the SDG goals presents a unique opportunity to apply a holistic, people-centred, and multisectoral approach to examine housing insecurity as an upstream determinant that shapes downstream health outcomes through cumulative and developmentally patterned processes [33,35,36]. Guided by this theoretical approach, this rapid review explores the impact of housing insecurity on health outcomes through a life-course theoretical framework, with this exposure during critical periods and across developmental trajectories contributing to persistent biological, psychosocial and environmental health disparities. This approach enables age-operationalized assessments, highlighting how structurally produced housing conditions shape health trajectories and contribute to long-term health disparities.

## 2. Methodology

**Figure 1:**
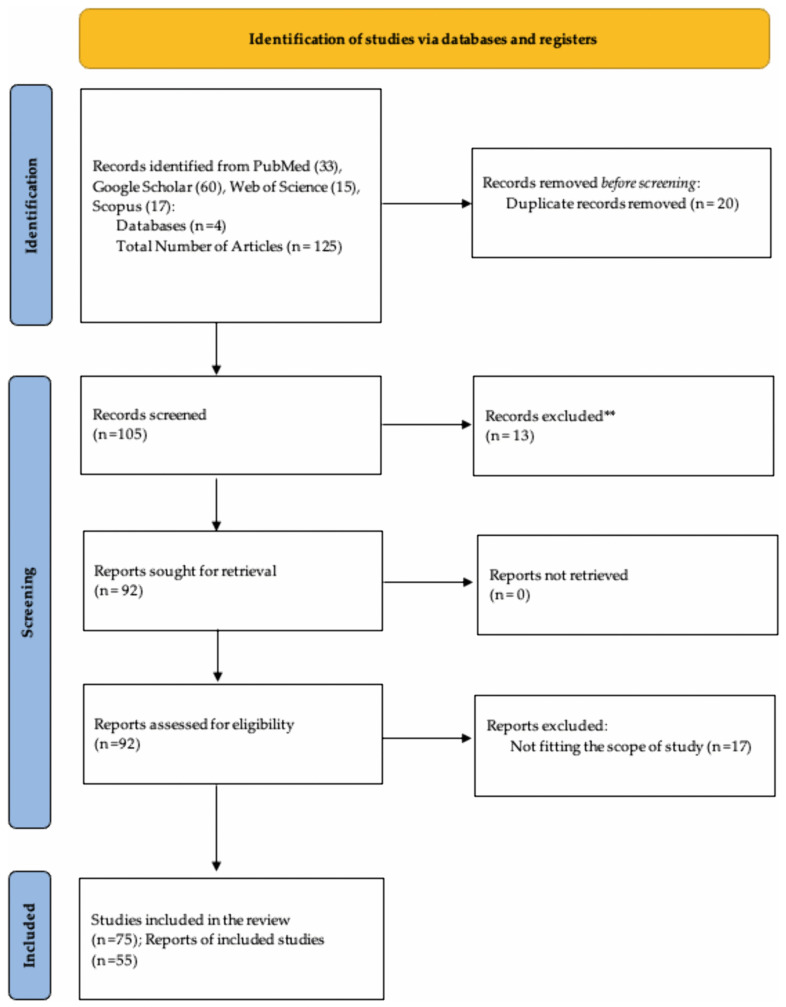
PRISMA Flow Diagram shows the study identification and selection process for the rapid review.

We conducted a rapid review exploring the health impacts of housing insecurity across the life course, starting from pregnancy, infancy, childhood, adolescence, adulthood, to the elderly. We have utilized peer-reviewed publications from 1991 to 2025 to ensure inclusion of recent scholarly literature. We searched PubMed, Google Scholar, SCOPUS and Web of Science for peer-reviewed publications. The key words included “housing insecurity”, “housing instability”, “housing crisis”, “health outcomes”, “infants”, “prenatal”, “childhood”, “adolescence”, “adulthood”, “elderly”, “ageing”, “life course perspective” and “life course theory”. These key terms ensured the location of relevant information to review. The inclusion criteria included articles which surround the topics of “epigenetics”, “life course theory”, “chronic illness” and “disease”, “biological markers”, as well as “climate change”. We applied a life course framework with age-stratified analysis, enabling the synthesis of findings within distinct developmental stages. Eligible studies were restricted to peer-reviewed publications in English that employed qualitative, quantitative or mixed-method approaches and reported measurable health outcomes associated with housing insecurity. The exclusion criteria included policy briefs solely focused on technical housing policy, political advocacy or economic modelling without reporting measurable health outcomes. This exclusion included commentaries, editorials, government reports, dissertations and non-peer-reviewed sources. The identification, screening, eligibility, and inclusion process for this rapid review is summarized in a PRISMA flow diagram (Figure 1).

## 3. Results

The search produced 125 articles from 4 search engines. This includes 33 articles from PubMed, 60 from Google Scholar, 17 from SCOPUS, and 15 from Web of Science. After the removal of duplicates and screening following the inclusion criteria, a total of 75 articles were eligible for review, with evidence from 55 studies included in the report. A synthesis of the articles has been developed to reflect the impact of housing insecurity at each stage of the life course (Table 1).

### 3.1. Housing Insecurity’s Effect on Health Outcomes for Pregnant Women

Evidence examining housing insecurity during pregnancy consistently demonstrates elevated risks for adverse maternal and infant health outcomes. Across studies, housing insecurity was associated with increased obstetric complications, poorer maternal health, and disrupted access to prenatal care [37,38,39]. These findings were observed across diverse populations and healthcare settings, underscoring pregnancy as a particularly vulnerable life stage for the health impacts of housing insecurity. Among pregnant women, housing insecurity heightens the risk of adverse pregnancy outcomes and long-term health deficits [37]. Homelessness during pregnancy was associated with increased likelihood of preterm delivery, with reduced gestation age, and higher rates of caesarean section compared with housed counterparts [38]. Caesarean sections impact both post-partum healing and subsequent pregnancies, with surgery increasing the likelihood of complications for homeless patients [38]. Housing insecurity was also linked to severe maternal morbidity during hospitalization, with housing-insecure patients exhibiting more than twice the odds of severe maternal morbidity relative to housing-secure patients [39]. Collectively, these findings indicate a consistent association between housing insecurity and heightened obstetric risk.

Mental health outcomes emerged as a prominent and recurrent pattern within evidence for this life stage. Housing insecurity during pregnancy was frequently associated with elevated psychological distress, including stress, anxiety, and depressive symptoms, often in the context of economic hardships [40,41]. Studies further linked maternal psychological distress to elevated infant cortisol levels, suggesting passive transmission of intergenerational health effects [41], programming fetal stress response systems. Housing insecurity during pregnancy creates intergenerational transmission pathways. It is also proposed that oxidative stress-induced DNA damage may disrupt DNA transcription, increasing the number of placental DNA adducts [42]. While the magnitude of these associations varied across studies, we highlight maternal mental health as a key pathway through which housing insecurity may influence both maternal and infant outcomes.

Additionally, housing insecurity was also associated with reduced access to prenatal and postnatal healthcare services. Homeless pregnant women were significantly less likely to initiate prenatal care during the first trimester and experienced greater barriers to health care access overall [43]. During the post-partum period, the risk of chronic conditions such as hypertension, cardiovascular disease, and declining health among mothers led to a greater likelihood of the mothers requiring intensive care and longer hospital stays [37]. This contributes to multifactorial exposures to undernutrition, food insecurity, psychosocial stress, and obstetric complications for maternal women [42]. These patterns suggest housing insecurity disrupts continuity of care during pregnancy and the immediate postpartum period, with implications for both short-term and longer-term health outcomes.

Environmental exposures further create health risks among housing-insecure pregnant women. Studies documented that increased exposure to air pollutants due to housing insecurity can affect fetal growth directly by passing across the placenta, worsening maternal health during pregnancy [42]. This perspective portrays maternal health as having unique health risk factors from early reproductive conditioning and psychosocial stressors across the life course [44].

Housing insecurity among pregnant women represents a breakdown in maternal vulnerability, marked by interpersonal stress and heightened perceived stress from life changes, daily hassles, and chronic strain, which contribute to parental stress affecting caregiving quality [45,46]. Thus, the life-course perspective of pregnancy represents a critical period during which housing insecurity can significantly exert health risk factors on both maternal health trajectories and future reproductive outcomes, reinforcing health inequities across generations.

### 3.2. Housing Insecurity Effect on Health Outcomes for 0−5-Year-Old Infants

Evidence from the review consistently demonstrated that housing insecurity during infancy is associated with adverse birth outcomes and heightened vulnerability to infectious and developmental health conditions. Across multiple studies, housing insecurity was associated with disrupted early-life health trajectories during a critical period for physiological, cognitive and immune development.

Several studies identified a strong association between housing insecurity and adverse birth outcomes. Housing insecurity was associated with a substantially increased risk of low birth weight and preterm birth, reporting a 73% higher risk among housing-insecure mothers [37]. Although gestational age did not differ largely between homeless and non-homeless mothers, at 38.6 weeks, the mean birth weight was significantly lower among infants born to homeless women (3242 g (7.1 lb) versus 3311 g (7.3 lb), *p* < 0.001) [47]. This indicates compromised fetal growth rather than shortened gestation, which produces a lower birth weight among neonates [47].

Housing insecurity was further associated with increased healthcare needs and barriers to preventive care during infancy. Infants born into housing-insecure households were 64% more likely to require admission to a neonatal intensive care unit (NICU) and had a 66% higher likelihood of extended hospitalization after delivery [37]. This increased the likelihood of unmet healthcare needs during a critical developmental window.

Additionally, environmental conditions emerged as a recurrent health risk factor for infectious disease and respiratory outcomes among infants. Poor housing quality, including overcrowding, inadequate ventilation and substandard sanitation [32], was linked to increased exposure to airborne and environmental pathogens [48,49]. Insufficient ventilation and overcrowded living spaces increase airborne pathogen concentration, while inadequate sanitation heightens the risk of respiratory infections and gastrointestinal infections among infants [48,49]. For instance, infants living in overcrowded households or sheltered environments were particularly vulnerable to mould, dust mites, tobacco smoke, insufficient ventilation systems, and unhygienic bedding [48]. These factors increase the risk of health outcomes such as tuberculosis infections and skin abrasions due to the proximity of infected individuals and immature immune systems of infants [48,49,50]. Housing insecurity also exacerbates poor nutrition having consequences such as delayed neural maturation and cognitive and motor deficits among neonates [51]. Signs include hypotonia (decreased tone), hyporeflexia, and motor nerve conduction delays [51]. These effects underscore the critical importance of stable housing for optimal infant development and health outcomes. Collectively, the evidence indicates that housing insecurity during the first five years of life has a significant association with health risks, including impaired fetal growth, increased medical utilization, reduced access to preventative care and heightened exposure to environmental toxins. From a life course perspective, the life stages of neonatal to infancy represents a sensitive period in which housing insecurity may disproportionately affect health trajectories early enough to have potential implications for development and disease vulnerabilities to be established in childhood.

### 3.3. Housing Insecurity on Health Outcomes During Early to Late Childhood (Ages 6−12 Years)

Evidence from the synthesized text indicates that housing insecurity during early to late childhood is associated with developmental and psychosocial health outcomes, reflecting the cumulative effects of prolonged exposure to unstable living conditions during critical periods of growth. Foundational pediatric research linked housing insecurity with lower weight-for-age and elevated developmental risk [52]. These outcomes are indicative of early biological embedding that may persist into later childhood [52].

Children from families that are housing insecure have a greater vulnerability to poor health conditions compared to peers with secure housing environments [52]. For example, sleep disturbances, unmet health needs, and increased exposure to environmental and social stressors, collectively contribute to physiological strain during childhood with higher anxiety and depression symptomatology [24]. In addition, elevated risk of child maltreatment was observed among housing-insecure families, exposure that is widely recognized as biologically consequential in the ability to induce stress-system dysregulation and long-term chronic illness [53].

During the childhood developmental period, psychological impacts of housing insecurity create social exclusion and problematic behaviour from instability due to lack of security and privacy [52,54]. Housing insecurity during middle childhood was associated with increased externalizing symptoms (e.g., aggression, defiance, hyperactivity), while issues related to internalizing symptoms (e.g., anxiety, depression, emotional withdrawal) emerged later [52]. Evidence suggests that these behavioural outcomes vary by duration and severity of exposure, and that prolonged exposure to housing burden and repeated instability has amplified adverse psychological effects compared to shorter, temporary hardship [53,54]. The link is largely mediated through increased parental psychological distress and parenting stress, reinforcing the need to address housing affordability to support early childhood psychological health [53,54]. Teachers and caregivers observed age-specific behavioural responses, with younger children more likely to exhibit withdrawal and separation anxiety, while older children exhibited increased anger and antagonistic behaviour [55].

Neurodevelopmental and cognitive outcomes further illustrate the health impacts of housing insecurity during this life stage. Housing instability and residential mobility were associated with poorer verbal cognitive abilities and academic performance, especially when instability occurs earlier in life [56]. This hinders their formative cognitive and psychosocial relations among their peers, further disrupting learning and increasing social isolation. The results from a structural equation model indicated that high levels of adverse childhood experiences (ACEs), including housing instability, were indirectly associated with poorer child neurodevelopmental functioning through increased caregiver stress affecting attention, emotional regulation, and social skills [55,57]. These findings suggest that housing insecurity influences child health through interconnected biological and psychosocial pathways that shape neurodevelopment during early and late childhood.

Altogether, the evidence indicates that housing insecurity between the ages of 5 to 12 is associated with biological outcomes such as impaired growth, developmental risk and health-related stress exposure, alongside behavioural and cognitive difficulties in young children. Childhood is a foundational stage to which early social, environmental and biological exposures can create change health trajectories for development but can be disrupted if housing insecurity is addressed promptly. Life-course perspective links this sensitive period to the consolidation of health exposures that can potentially extend into adolescence.

### 3.4. Housing Insecurity’s Effect on Health Outcomes for Adolescents (Ages 13−17 Years)

We found significant evidence for the health impacts of housing insecurity during adolescence, a developmental period marked by continued biological maturation alongside increasing cognitive and social independence [58]. Exposure to substandard housing or unstable housing conditions was associated with poorer self-rated health and an increased risk of cardiometabolic conditions and obesity [59,60]. Longitudinal evidence further suggests that repeated exposure to housing insecurity during adolescence was also associated with elevated inflammation levels, measured by C-reactive protein (CRP), indicating increased physiological stress and heightened risk for chronic diseases such as cardiovascular disease later in life [61]. Notably, exposure around mid-adolescence (approximately age 14) appeared to represent a sensitive period for these biological effects, coinciding with heightened social transitions [61].

Psychosocial and behavioural outcomes were consistently reported across the literature. Housing insecurity in early childhood and adolescence was associated with higher levels of anxiety, depressive symptoms and behavioural problems in adolescence [62]. Longitudinal analysis demonstrates that early housing insecurity predicted emotional and behavioural difficulties in adolescence, including aggression and internalizing symptoms [62]. These associations persisted after accounting for SES factors as they were partly mediated by caregiver stress and psychologically aggressive parenting, indicating that housing insecurity shapes adolescent mental health through family-level stress processes [62,63,64]. Adolescents exposed to housing instability were also more likely to engage in health-risk behaviours, including smoking, unhealthy eating patterns, which further contribute to adverse health trajectories into adulthood [59]. In addition, mental health challenges, including higher rates of depression, suicidal ideation, and perceived stress, were found to be prevalent at this life stage [59].

Collectively, the evidence indicates that adolescence represents a pivotal life-course stage in housing insecurity associated with the consolidation of biological, psychosocial and behavioural health risks. Across multiple studies, exposure to housing insecurity during this period was linked to emerging chronic disease risk, persistent mental health challenges, and health-related behaviours that extend into adulthood. These findings suggest that housing conditions during adolescence shape health trajectories at a critical juncture between childhood vulnerability and adult disease onset.

### 3.5. Housing Insecurity Effect on Health Outcomes During Early to Late Adulthood (Ages 18−55)

Adulthood represents a critical convergence point where exposures and experiences from earlier life stages manifest as measurable health decline. During this period, the social, behavioural, and biological consequences of past predictors of childhood and adolescence become most apparent during adulthood, reflecting the trajectory of an individual’s life course.

Housing insecurity in adulthood creates reduced access to healthcare and higher rates of acute and chronic illnesses. Adults experiencing housing insecurity face systematic exclusion from preventative care: they are significantly more likely to lack a usual source of care, forgo routine check-ups, and delay necessary medical treatment due to cost burdens created by housing expenses [64]. These patterns highlight significant disparities in healthcare access among housing-insecure populations by preventing early detection and management of emerging health conditions [64]. Consequently, housing-insecure adults report higher numbers of chronic diseases compared to their stably housed counterparts [64]. These patterns reveal how housing instability contributes to disease burden not only through direct exposure but also through disruption of healthcare continuity during adulthood.

Beyond chronic conditions, housing insecurity in adulthood is strongly associated with cumulative exposure to infection risk through both behavioural adaptations and environmental exposures. Housing insecurity increases exposure to high-risk sexual behaviours, including transactional sex and reduced condom use, thereby elevating the prevalence of STIs, specifically HIV and Hepatitis C [65]. This vulnerability intensifies among people who inject drugs, where the combination of unstable living environments and reduced access to preventive care services creates compounding infection risk [65]. These findings demonstrate how housing insecurity operates as a structural determinant that shapes individual-level health behaviours under conditions of constrained agency.

Consistent with patterns observed earlier in the life course, mental health challenges persisted and often intensified during adulthood among individuals experiencing housing insecurity. Adults exposed to unaffordable or multiple housing instability measures exhibited a higher prevalence of depression and anxiety [66,67]. Importantly, these mental health effects represent the accumulation of psychosocial stress across developmental transitions into employment, financial independence and family formation during working-age years [66,67]. Housing instability creates a dual burden: immediate psychological distress combined with derailed life trajectories that compound health disadvantages over time [67]. These mental health outcomes represent the continuation and amplification of vulnerabilities established earlier in life.

Housing insecurity during adulthood activates physiological and epigenetic manifestations of stress among aging adults, creating a proximal-to-distal stress cascade [68]. Chronic housing insecurity was associated with sustained activation of the sympathetic-adrenal-medullary (SAM) and hypothalamic-pituitary-adrenal (HPA) axes, producing acute stress responses including cortisol spikes and heart rate variability [68]. However, sustained exposure transforms acute responses into chronic physiological dysregulation. Over time, persistent reactivity leads to allostatic load–a cumulative “wear and tear” across bodily systems–and drives epigenetic modifications that alter gene expression patterns [68]. These distal manifestations represent biomarkers of stress-related aging and create lasting vulnerability to chronic disease [69]. For instance, poor housing conditions produce acute deterioration in mental health with downstream physical health implications, which are mediated through epigenetic mechanisms [70]. This establishes a pathway where mental health decline triggers biological changes that, in turn, increase physical disease susceptibility [69,70]. These findings reveal that housing insecurity is a fundamental determinant that can potentially reshape physiology across multiple systems.

The physiological impact of housing insecurity does not affect all adults equally, as structural discrimination operates as a powerful modifier that amplifies biological harm. A study demonstrates that adults experiencing both housing insecurity and housing-related discrimination face compounded stress physiology beyond additive effects, introducing the term “double jeopardy” [68,71]. Historic and ongoing housing-related discrimination– including redlining, racial steering and age-based eviction practices–intensifies the physiological burden of housing insecurity through three interconnected pathways [68,71]. First, discrimination reduces access to structural supports, limiting opportunities to secure safe neighbourhoods and stable housing that might buffer stress effects [67]. Second, perceived discrimination directly worsens health behaviors including sleep quality and substance use patterns, creating additional physiological strain [71]. Third, discrimination undermines psychosocial resources by eroding social support networks and mental wellness, removing protective factors that might otherwise moderate stress responses [71]. Racialized adults with intersecting vulnerabilities, such as disabilities, experience deeply rooted health inequities stemming from lifelong structural oppression, including segregation in housing, limited educational and employment opportunities, and increased chronic disease risk [9]. Black and Hispanic adults show disproportionately higher rates of hypertension and diabetes compared to White adults, disparities reflecting accumulated exposure to inadequate housing and systemic inequities rather than individual-level factors [9]. Gender identity intersects with race and class to create unique vulnerabilities to housing insecurity among transgender populations. Housing instability among transgender adults was associated with elevated stress, mental health challenges, barriers to employment, and difficulty accessing healthcare, demonstrating how discrimination-driven housing insecurity operates through multiple interconnected pathways to undermine health [72]. Moreover, housing insecurity operates through epigenetic mechanisms to program health trajectories that extend across the remaining life span. Minority and older adults who experienced intersecting housing insecurity and discrimination exhibit greater allostatic load, display inflammatory gene expression patterns, and demonstrate accelerated epigenetic aging markers [67,71]. These epigenetic changes represent biological scarring from housing insecurity that persists even after housing conditions improve, increasing vulnerability to disease, stress reactivity, and aging processes throughout later life [71].

Unlike infancy or adolescence, where the emphasis is on sensitive periods, this life stage illustrates how chronic exposure to housing insecurity becomes biologically embedded [68]. Moreover, the life course perspective reveals adulthood as a critical period not due to initiation of health vulnerability, as discussed in the previous life stage findings, but the life stage where earlier exposures fully manifest in health outcomes. Housing insecurity operates in adulthood as both an outcome of cumulative health disadvantage and a determinant of accelerated aging and disease in the decades to follow.

### 3.6. Housing Insecurity Effect on Health Outcomes During Old Age (Ages 55+)

Old age represents the culmination of life-course exposures to housing insecurity, where decades of accumulated disadvantages converge with intrinsic aging processes to create unique and amplified health vulnerabilities. Unlike younger life stages where housing insecurity initiates or accelerates health decline, in older adulthood it interacts synergistically with age-related physiological deterioration—including immune senescence, frailty, sensory impairments, and medication burden—to compound health risks beyond the sum of individual factors [73]. Longitudinal evidence demonstrates that housing insecurity effects persist even after controlling for prior health status, indicating these are enduring, structural determinants rather than consequences of pre-existing poor health [32].

Housing insecurity during older adulthood produces measurable acceleration of biological aging through epigenetic pathways. A study examining the relationship between housing circumstances and epigenetic aging found that challenging housing conditions are associated with accelerated biological aging [74]. This acceleration is measurable through epigenetic clocks, which assess DNA methylation patterns to estimate biological age–revealing that individuals experiencing unstable or inadequate housing may undergo faster biological aging, potentially leading to the earlier onset of age-related diseases [74]. This includes cardiovascular disease, dementia, and metabolic disorders [74]. Next, epigenetic mechanisms serve as intermediaries between environmental exposures and individual development. A pilot study examined the association between socioeconomic status, which is strongly associated with housing insecurity and its correlation with altered DNA methylation, which affects stress regulation capacity [75]. Importantly, these molecular changes establish a link between housing conditions and heightened incidence of mental illness, lower self-rated physical health, and increased chronic disease burden consistently observed among housing-insecure older adults [70,76,77].

Next, housing insecurity in old age highlights the interactions between environmental hazards and age-related physical vulnerability [78]. Intrinsic aging impairments such as balance loss, visual and auditory decline, medication side effects, and frailty, transform unsafe housing conditions common under housing insecurity into severe injury risks that would pose minimal threat to younger adults [73]. Environmental hazards, including structural defects, poor lighting, absence of safety features such as grab bars, and unsafe bathroom conditions, interact synergistically with age-related physical decline to substantially elevate fall risk and serious injury [73,78]. Critically, evidence suggests that the physiological and functional damage accumulated during periods of housing insecurity may create lasting vulnerability that persists even after housing conditions improve. Research among formerly homeless older adults who achieved housing stability through permanent supportive housing demonstrates continued elevated fall incidence, with risk strongly associated with persistent frailty, functional impairment, and chronic pain [79]. Nearly half of falls occurred inside the home, particularly in bathrooms, indicating that environmental awareness alone does not eliminate risk when physiological damage has already embedded [79]. This pattern reveals that housing insecurity is a compounding burden of physical harm, including accelerated frailty, untreated chronic pain, and functional decline, that does not immediately resolve upon re-housing. The finding underscores the critical importance of early intervention to prevent the accumulation of irreversible physiological damage during periods of housing instability.

Environmental hazards extend beyond injury risk to include chronic respiratory threats. Living in damp, poorly ventilated, or mould-infested dwellings increases susceptibility to respiratory infections and chronic lung conditions among older adults, particularly when combined with age-related immune decline [80,81]. This environmental-biological interaction exemplifies how housing insecurity multiplies harm: substandard conditions that might cause minor discomfort in younger adults have serious and potentially fatal respiratory disease in older populations with compromised immune function.

Housing insecurity among older adults reflects gendered life-course trajectories, where accumulated disadvantages across decades manifest as housing vulnerability in later life. A study traced how women aged 60+ in Nova Scotia experienced housing insecurity as an outcome of lifelong structural inequities: earlier exposure to poverty, unstable employment, and gendered wage gaps systematically limited savings and asset accumulation, producing housing precarity decades later [82]. These life-course disadvantages interact with age-related health decline, making the biological effects of poor housing more severe [80,81,82]. This life stage also presents unique dimensions of safety concerns, trauma and coping mechanisms as survival strategies, while social isolation and environmental hazards further erode quality of life [80,81,82]. This behaviour underscores the complex interplay between housing insecurity and health-seeking behaviours.

The mental health impacts of housing insecurity in older adulthood operate through interconnected pathways linking psychological distress, social isolation, and nutritional deprivation. The stress of unaffordable or unstable housing generates or exacerbates mental health challenges, including anxiety and depression, which in turn increase risk of social isolation and loneliness—particularly concerning age-related shrinking of social networks [77]. However, the causal pathways warrant additional investigation to fully characterize these relationships [77]. What emerges is the clustering of vulnerabilities: nutritional deficits from food insecurity consistently co-occur with heightened anxiety and depressive symptoms among housing-insecure older adults [83]. This co-occurrence suggests shared causal pathways where housing instability simultaneously undermines both nutritional access and psychological well-being, creating synergistic harm that exceeds the impact of either factor alone [83]. The interconnection underscores the necessity of holistic interventions that address housing affordability, nutrition security, mental health support, and social connection simultaneously rather than treating these as independent domains.

Also, older adults experiencing housing insecurity face compounded disadvantage through intersecting forms of discrimination that operate across multiple levels. Older populations identify that structural, interpersonal and institutional ageism creates age-based social stigma while limiting access to housing and support services [83]. This discrimination operates through multiple mechanisms: elder abuse, social support erosion, and marginalization from services designed for younger homeless populations [83,84]. The discriminatory effects materialized through constrained resources. Older adults in social housing reporting food insecurity were significantly more likely to be underweight and living in poverty, revealing how housing insecurity directly channels into nutritional deprivation among older populations [83,84]. Housing instability creates economic strain that forces impossible trade-offs between housing costs and food purchases, resulting in undernutrition that accelerates frailty and disease progression [83,85].

Furthermore, housing insecurity systematically disrupts healthcare access among older adults through forced priority hierarchies where basic survival needs supersede medical care. Older adults facing housing instability consistently prioritize securing shelter and food over seeking medical treatment, leading to substantial unmet health needs and progressive worsening of manageable chronic conditions [32,82]. Older adults experiencing long-term homelessness or housing insecurity carry disproportionately high rates of chronic hepatitis C infection, typically acquired earlier in adulthood, but remaining untreated due to decades of limited healthcare access [86]. These chronic infections compound other age-related health challenges while remaining manageable with appropriate treatment, illustrating how housing insecurity transforms treatable conditions into sources of cumulative morbidity through systematic healthcare exclusion.

The use of the life course theory unveils how housing insecurity operates simultaneously and interdependently, calling for interventions that address housing stability, environmental safety, nutritional security, healthcare access, and social support as integrated components rather than isolated targets [84]. The persistence of physiological vulnerability even after re-housing emphasizes that preventing housing insecurity is far more effective than attempting to reverse its accumulated biological damage, underscoring the critical importance of early life-course intervention [79,84].

## 4. Discussion

This rapid review synthesizes evidence demonstrating that housing insecurity operates as a fundamental social determinant of health across the entire life-course, from pregnancy through older adulthood (Figure S1). Our findings reveal three core mechanisms through which housing insecurity shapes health trajectories: (1) biological embedding through physiological stress systems and epigenetic modifications, (2) disruption of critical developmental transitions at sensitive periods, and (3) cumulative burden accumulation that compounds vulnerability over time. Unlike previous research examining housing and health at isolated life stages, this life-course framework illuminates how exposures and vulnerabilities cascade across developmental periods, creating interconnected pathways from early adversity to late-life disease. The evidence demonstrates that housing insecurity is not merely correlated with poor health outcomes but actively programs biological systems, disrupts social trajectories, and embeds disadvantage in ways that persist across decades and potentially transmit intergenerationally.

### 4.1. Biological Embedding: Changes to Lifelong Vulnerability

A central finding across all life stages is that housing insecurity becomes biologically embedded through measurable physiological and epigenetic mechanisms. During pregnancy, maternal stress levels from housing insecurity elevates cortisol levels that cross the placenta, resulting in elevated infant cortisol levels, programming fetal stress response systems and establishing vulnerability that manifests as elevated infant cortisol and immune function [40,41]. This represents the earliest form of biological embedding, where social conditions closely reshape developing physiological systems before birth. The biological embedding process continues and intensifies throughout childhood and adolescence. During infancy, malnutrition resulting from housing insecurity produces structural brain changes, delayed neural maturation, and nerve conduction abnormalities that become neurologically “hardwired” [87]. By childhood, housing insecurity correlates with elevated inflammatory markers [54], and during adolescence, exposure around age 14 is associated with increased C-reactive protein levels—a biomarker of inflammation and chronic disease risk [61]. These findings reveal a progressive pattern where housing insecurity at each developmental stage leaves distinct biological signatures that accumulate over time. Surprisingly, adulthood represents a critical phase where the cumulative effects of earlier housing instability fully emerge, reflecting decades of biological wear and tear. In adulthood, the biological embedding mechanisms shift from establishing vulnerability to manifesting as measurable disease processes [69]. Housing insecurity activates chronic physiological stress through the sympathetic-adrenal-medullary (SAM) and hypothalamic-pituitary-adrenal (HPA) axes, producing sustained cortisol elevation and cardiovascular reactivity [68]. Over time, this persistent activation accumulates as an allostatic load—the cumulative “wear and tear” across multiple body systems—and drives epigenetic modifications that alter gene expression patterns [68,71]. These epigenetic changes, including DNA methylation of stress-regulatory genes, represent biological aging signatures that accelerate disease onset and create vulnerability persisting even if housing conditions later improve [70,74,75].

Housing insecurity in older adulthood acts as a compounded health risk multiplier, accelerating biological aging, amplifying chronic disease burden, and embedding health disadvantage. Older adults experiencing housing insecurity show faster epigenetic “clocks” (DNA methylation patterns) that predict earlier onset of age-related diseases, including cardiovascular disease, dementia, and metabolic disorders [74]. This acceleration reflects decades of accumulated physiological stress that has progressively reshaped biological systems. Critically, evidence from formerly homeless older adults demonstrates that even after achieving housing stability, elevated health risks persist—including continued high fall rates associated with frailty and functional impairment [79]. This reveals that biological damage from housing insecurity creates lasting vulnerability extending beyond the period of instability itself, underscoring the importance of prevention over remediation.

The biological embedding findings collectively demonstrate that housing insecurity operates through mechanistic pathways linking social conditions to physiological processes. This shift understanding of housing as a health determinant from theoretical observation to mechanistic explanation, with measurable biomarkers (cortisol, inflammatory markers, epigenetic signatures, allostatic load) providing evidence of how unstable housing intimately reshapes biological health trajectories across the lifespan.

### 4.2. Sensitive Periods and Developmental Timing

The life-course evidence reveals that the timing of housing insecurity exposure critically shapes health outcomes, with certain developmental periods demonstrating heightened vulnerability. Infancy emerges as a foundational sensitive period where housing mobility produces particularly severe impacts on development. This stage reveals several critical and underexplored insights into the intersection of housing insecurity, infant health, and life course risk accumulation. First, the evidence for biological embedding from prolonged malnutrition due to housing insecurity in early infancy is linked to measurable structural brain changes, delayed neural maturation, and nerve conduction abnormalities, suggesting that disadvantage becomes neurologically “hardwired” in ways that may persist throughout life [87]. The finding that mobility during infancy creates worse cognitive outcomes than mobility during later childhood suggests early neural development windows where environmental stability is especially critical for optimal brain maturation [56]. Adolescence represents a second critical sensitive period, though operating through different developmental mechanisms than infancy. The convergence of biological changes (puberty, brain development) with social transitions (educational progression, identity formation, peer relationships) creates a period where housing instability simultaneously disrupts multiple developmental domains [58,62]. The evidence shows temporally distinct impacts: externalizing symptoms (aggression, defiance) emerge progressively during housing instability, while internalizing symptoms (anxiety, depression) often appear later, suggesting a delayed psychological toll that manifests after prolonged housing insecure exposure [54,56,59]. Additionally, exposure to housing insecurity around age 14 associates with elevated inflammatory markers that do not appear as consistently at other ages, indicating a biological sensitivity window during mid-adolescence [61].

However, the sensitive period concept requires nuance: while certain developmental windows show heightened vulnerability, cumulative exposure across multiple life stages produces even stronger effects than single-period exposure. Housing insecurity persisting from childhood through adolescence creates a greater mental health burden than instability confined to one developmental stage [24]. This suggests both critical periods of acute sensitivity and cumulative risk models, where repeated or prolonged exposure compounds harm over time. The temporal patterns also reveal important dynamics in symptom manifestation. Behavioural responses to housing insecurity in childhood show fluidity, with children oscillating between withdrawal and antagonistic behaviour rather than maintaining static emotional states [55]. Similarly, mental health impacts often emerge with delay: housing insecurity during childhood predicts anxiety and depression in both adolescence and adulthood, even years after housing conditions stabilize [24]. These findings challenge linear models of exposure and outcome, revealing housing insecurity effects as dynamic processes that unfold across developmental trajectories. Understanding sensitive periods has intervention implications: preventing housing instability during infancy and adolescence may yield disproportionate health benefits, while interventions during these windows might more effectively interrupt developing pathways toward chronic disease. However, the cumulative exposure findings equally emphasize that sustained housing insecurity across childhood and adolescence remains critical, as repeated instability compounds risk regardless of when it occurs.

### 4.3. Cumulative Burden and Intergenerational Transmission

A defining characteristic of housing insecurity’s health impacts is cumulative burden—the progressive accumulation of risk exposures and health vulnerabilities across the life course. This operates through multiple pathways that interconnect and amplify over time. During pregnancy, housing insecurity increases maternal stress, reduces prenatal healthcare access, and elevates exposure to environmental toxins—establishing fetal vulnerability before birth [37,40,42]. Infants born into housing-insecure circumstances show increased risk of low birth weight, preterm birth, and NICU admission, creating early health deficits that influence subsequent developmental trajectories [37].

These early vulnerabilities show progression: infants experiencing housing insecurity attend fewer well-child visits and face greater infectious disease risk from poor housing conditions [48,49]. During childhood, housing instability disrupts education through school mobility, which compounds behavioural challenges through parental stress, and social isolation [56,57,61]. By adolescence, these accumulated disadvantages manifest as mental health challenges, risky health behaviours (smoking, unhealthy eating), and inflammatory biomarkers predicting future chronic disease [59,60,61]. The cumulative burden becomes fully apparent in adulthood, where housing insecurity correlates with multiple chronic diseases, reduced healthcare access, elevated infectious disease risk, and mental health disorders [64,65,66]. Critically, these adult health burdens reflect not only current housing conditions but also the accumulated physiological and social damage from earlier life stages. The progression from infant cortisol elevation to childhood inflammation to adolescent behavioural changes to adult chronic disease illustrates how housing insecurity at each stage establishes vulnerability for the next, creating cascading health decline across decades. Older adulthood represents the culmination of this cumulative process, where decades of housing-related stress converge with age-related physiological decline. Older adults experiencing housing insecurity show a greater burden of chronic conditions even after controlling for prior health status [32], indicating housing effects that persist and compound beyond what earlier health alone would predict. The accelerated epigenetic aging observed among housing-insecure older adults [74], provides evidence of cumulative wear on biological systems accumulated across the lifespan.

The cumulative burden framework also illuminates intergenerational transmission pathways. The intergenerational transmission of health risks linked to housing insecurity highlights how social determinants do not just affect individuals in isolation but cascade through familial generations over time [44,88]. Maternal stress from housing insecurity is apparent in fetal physiology through elevated cortisol, establishing vulnerability that can extend into the next generation [26,37,38,40,41]. Parents experiencing housing insecurity demonstrate increased psychological distress and altered parenting quality, affecting children’s emotional development and attachment patterns [54]. This creates cycles where housing-insecure parents transmit both biological vulnerabilities (through prenatal programming) and social vulnerabilities (through disrupted caregiving) to their children, who then face elevated risk of housing insecurity in their own adulthood due to accumulated educational, economic, and health disadvantages. Breaking these intergenerational cycles requires interventions targeting both immediate housing stability and the accumulated vulnerabilities transmitted across generations. This suggests multigenerational support approaches, including stable housing provision, mental health services, healthcare access, and community resources designed to interrupt cascading disadvantage before it embeds across family lineages.

### 4.4. Structural Discrimination in Housing Insecurity

Throughout the life course, structural discrimination amplifies housing insecurity’s health impacts, creating differential vulnerability across social groups. This operates as an effect modification: housing insecurity produces worse health outcomes among populations simultaneously experiencing discrimination based on race, age, or gender. During adulthood, the evidence demonstrates this “double jeopardy” most clearly. Adults experiencing both housing insecurity and chronic discrimination—including historical practices like redlining and ongoing racial steering—show greater allostatic load, increased inflammatory gene expression, and accelerated epigenetic aging compared to those experiencing housing insecurity alone [67,68,71]. The discrimination effects operate through three interconnected pathways. First, discrimination reduces access to structural supports, including safe neighbourhoods and stable housing that might buffer housing insecurity’s physiological impacts [71]. Second, discrimination worsens health behaviors through the denial of housing, including sleep quality and substance use, adding additional physiological strain [71]. Third, discrimination undermines psychosocial resources by eroding social support networks and mental wellness, removing protective factors that might otherwise moderate stress responses [71]. These pathways create multiplicative rather than additive harm, where discrimination magnifies housing insecurity’s biological embedding beyond what either exposure alone would produce. Among older adults, ageism operates as a powerful discriminatory force intersecting with housing insecurity [83,84]. Structural ageism limits access to housing services designed primarily for younger populations, while interpersonal ageism creates social stigma, reducing help-seeking [83,84].

The discrimination findings reveal housing insecurity as fundamentally rooted in structural inequality. The overrepresentation of racial minorities among housing-insecure populations reflects historical injustices including residential segregation, discriminatory lending, and exclusionary zoning [9,10]. Folorunsho (2025) demonstrates that early-life disadvantages—including growing up in underserved neighbourhoods and experiencing systemic discrimination—compound across the lifespan, establishing trajectories toward chronic health conditions and financial insecurity visible in adulthood [9]. The resulting health disparities, including disproportionately higher rates of hypertension and diabetes among Black and Hispanic adults compared to White adults, reflect not individual choices but structural inequities operating [9]. The intersectional component is essential for understanding how multiple marginalized identities combine to shape housing insecurity’s health impacts. Among transgender populations, gender identity interacts with race, class, and physical presentation to create unique vulnerability that differ from cisgender populations [9]. This demonstrates that housing insecurity cannot be understood through single-axis analyses of race or gender alone but requires recognition of how intersecting aspects of identity interact to produce differential exposure and health impacts.

These systemic forces shape both who experiences housing insecurity and how severely it impacts health along lines of race, gender, and age. Addressing housing insecurity, therefore, requires confronting the structural discrimination that produces differential exposure and vulnerability. Interventions providing housing alone, without addressing the discriminatory systems that created housing instability, risk perpetuating inequities even while improving individual housing situations.

### 4.5. Implications for Intervention and Policy

The life-course evidence reveals multiple intervention points and approaches for reducing housing insecurity’s health impacts. For example, nearly one-third of Americans spend 30% or more of their household income on housing, with 83.4% of households earning under $20,000 annually paying more than 30% toward housing costs [89]. This affordability crisis creates systematic trade-offs between housing, healthcare, and food security [89]. Thus, housing is a fundamental health infrastructure rather than merely a social concern.

Early life interventions preventing housing instability during pregnancy and infancy could interrupt biological embedding processes before they establish lasting vulnerability. The life-course evidence demonstrates that housing instability during sensitive periods—particularly pregnancy, infancy, and adolescence—establishes vulnerability cascading across decades, producing both immediate health impacts and long-term effects on education, employment, and income trajectories [25]. Prevention during these windows interrupts biological embedding before it becomes irreversible, making early intervention far more effective and cost-efficient than attempting to reverse accumulated physiological damage in adulthood. This suggests prioritizing housing stability for pregnant women and families with young children as a primary prevention strategy through rental assistance programs, eviction prevention policies, and integration of housing screening into prenatal and pediatric healthcare settings [90].

During childhood and adolescence, interventions must address both housing stability and the accumulated health and developmental impacts from earlier instability. School-based identification of housing risk before homelessness develops represents a critical prevention opportunity, as school suspensions and other disciplinary actions predict later housing insecurity [91]. This requires combining housing provision with educational support, addressing school mobility effects, mental health services treating emerging anxiety and depression, and family support reducing parental stress affecting child outcomes. Healthcare settings should systematically screen for housing instability as part of routine pediatric and adolescent care, enabling early intervention [90].

For adults, the evidence supports multiple integrated intervention approaches addressing the complex interplay between housing insecurity, healthcare access, chronic disease management, and mental health. Primary care-based housing support programs reduce outpatient visits by 2.5 annually while patients report mental and physical health improvements attributed both to obtaining housing and to advocates’ compassionate support [92]. These findings indicate that healthcare systems can effectively address housing instability when screening, referral, and support services are integrated into clinical care [90,92].

However, systematic review evidence reveals important limitations in current intervention approaches. While Housing Choice Voucher Programs show consistent evidence of reduced overcrowding, food insecurity, and concentrated poverty exposure [93], overall evidence for housing interventions improving adult health outcomes remains mixed and mostly of low certainty, with the evidence found for eviction moratoriums [94]. This suggests existing interventions may need pairing with other policy efforts addressing structural determinants of health rather than focusing solely on individual housing placement [94]. Simply providing housing—while necessary—may be insufficient to reverse the accumulated biological and social damage from prolonged housing instability documented throughout this review. The biological embedding, cumulative burden, and structural discrimination mechanisms identified across the life course indicate that housing provision alone cannot address the physiological dysregulation (allostatic load, epigenetic aging), disrupted educational and employment trajectories, accumulated trauma, and social isolation that develop during periods of housing insecurity. In other words, once individuals have experienced years of housing instability, merely securing stable housing does not automatically undo the health harm already embedded. Effective responses require multi-level strategies: Direct provision (immediate level) encompasses the proximate ways to ensure survival needs are met through rental assistance [95]. Partner (cross-sector collaboration) recognizes that housing intervention must integrate a multidisciplinary approach of healthcare, education and social support systems to address accumulated deficits [95]. Invest (long-term structural change) requires addressing structural inequities through transformative systems that produce housing insecurity [95]. This strategy acknowledges that addressing housing insecurity requires simultaneous attention to immediate needs, collaborative service integration, and fundamental policy reforms, as long-term solutions require transforming systems. We cannot intervene our way out of structural-level challenges using individual-level solutions.

Housing stability is a foundational public health investment. The evidence that housing insecurity at each life stage establishes vulnerability for subsequent life stages suggests that preventing housing insecurity early yields compounding benefits. This supports significant public investment in affordable housing, rental assistance, and eviction prevention pursuits, as possible cost-effective health interventions to prevent expensive downstream chronic disease management and well-being. Achieving population health and reducing health inequities requires recognizing housing as essential health infrastructure and prioritizing housing stability across the life course as a primary prevention strategy.

### 4.6. Knowledge Gaps

Using a life course framework to assess the evidence of housing insecurity and health outcomes, this rapid review identified several important gaps in the literature. Across life stages, there is limited studies examining the health effects of housing insecurity during infancy, late childhood and adolescence. In addition, the evidence base is heavily skewed toward high-income countries, with relatively few studies conducted in Low- and Middle-Income Countries, limiting the global generalizability of findings. Next, substantial heterogeneity was observed in how housing insecurity or instability was conceptualized and measured across studies, underscoring the need for standardized, cross-culturally validated measures. Finally, few studies conducted subgroup analyses pointing to a critical gap in understanding how housing insecurity intersects with social stratifiers such as race/ethnicity and indigeneity, sex and gender, place of residence, or income and wealth to shape health outcomes across the life course.

### 4.7. Strengths and Limitations

The primary strength of this review lies in applying a life-course framework to synthesize housing insecurity’s health impacts across all developmental stages. This approach reveals patterns invisible when examining isolated life periods, including biological embedding mechanisms, sensitive period effects, cumulative burden accumulation, and intergenerational transmission pathways. Unlike previous research examining housing and health at single developmental stages, this life-course synthesis illuminates how exposures during early developmental windows create lasting biological changes with repercussions extending through late-life transitions, establishing housing stability as a foundational determinant whose impacts compound across the lifespan to shape health trajectories and perpetuate health inequities. A comprehensive public health approach that integrates economic, social, and environmental factors is critical to mitigating health inequities and improving long-term health outcomes. Nonetheless, this study has some limitations. First, using a rapid review means the search may not have been as comprehensive, implying there is a higher risk that this review missed some existing studies that focus on housing insecurity. Second, the use of rapid review reduces the ability to reproduce the same findings, which calls for future reviews to consider using systematic reviews for a more comprehensive approach. Thirdly, unlike systematic reviews, rapid reviews do not have universally accepted standards for assessing their quality, although we have taken appropriate measures to ensure rigour. We employed a rapid review methodology rather than a systematic review due to the exploratory nature of this research. No previous review has examined housing insecurity’s health impacts across the entire life course using a life-course theoretical framework. Rapid reviews are appropriate for scoping broad topics, identifying knowledge gaps, and establishing conceptual frameworks before conducting more focused systematic reviews [96,97]. This approach allowed us to map the breadth of evidence across developmental stages, identify mechanistic patterns (biological embedding, sensitive periods, cumulative burden), and highlight areas requiring deeper investigation. The findings establish a foundation for future stage-specific systematic reviews examining particular mechanisms or populations in greater depth. While this methodology enabled broad synthesis and pattern identification across the life course, it carries limitations, including potentially incomplete literature capture and reduced reproducibility compared to full systematic reviews.

## 5. Conclusions

Housing insecurity is both a fundamental determinant of health equity and a structural barrier to human agency across the lifespan. The findings demonstrate that housing insecurity is not simply correlated with poor health outcomes—it operates as a cumulative burden that transforms across developmental stages, from biological programming before birth to accelerated aging in later life, creating chronic health disparities that cascade through generations. Housing insecurity creates resource scarcity so profound that healthcare becomes a luxury rather than a necessity, forcing trade-offs between securing shelter and accessing medical care, adequate nutrition, and other basic needs essential to health. This is not merely an economic problem but a crisis of health equity. The life-course framework employed here reveals how housing insecurity amplifies the magnitude of acute and chronic exposures to adversity at each life stage, requiring recognition of multilevel adaptations and responses across these intersecting systems. To address the cumulative effects of housing insecurity, health equity must become central to urban governance and planning. The persistence of what effectively functions as urban slums within metropolitan cities—characterized by substandard housing quality, environmental hazards, inadequate infrastructure, and systematic neglect—represents a failure to prioritize the conditions in which individuals live, grow, and age as fundamental drivers of health outcomes. Affordable housing must therefore be recognized not as one policy priority among many but as foundational to any meaningful agenda for improving health equity. Addressing housing insecurity requires confronting the structural determinants—the causes of the causes—that shape who experiences housing instability and under what conditions. Economic development alone cannot serve as a curative “prescription for poverty.” While economic solutions may address certain dimensions of health disparities, they cannot dismantle the deeply embedded social, political, and institutional barriers that perpetuate housing inequities. Interventions addressing only proximal risk factors—clinical treatment of disease, behavioral health counselling, educational support—while ignoring the fundamental social determinant of housing stability will perpetuate rather than resolve health inequities. Housing insecurity, examined through a life-course lens, reveals the profound interconnection between where people live and how they live—and ultimately, how long and how well they live. The patterns documented here demonstrate that stable housing is not simply shelter but foundational infrastructure for health, development, and human agency across the lifespan. The life course framework makes clear that the costs of inaction compound across lifetimes and generations, while the mechanisms of biological embedding and intergenerational transmission demonstrate that current housing instability creates health vulnerabilities extending far into the future. Addressing this requires transforming not only housing policy, but the broader structural conditions that determine who experiences housing insecurity and why, moving from managing the symptoms of inequality to dismantling the systems that produce it is quintessential.

## Figures and Tables

**Table 1:** Pathways Linking Housing Insecurity (Instability) to Biological, Psychosocial, and Environmental Health Outcomes Across the Lifespan.

Life Stage	Biological Outcome	Psycho-Social Outcome	Environmental Outcome
Pregnancy (in utero)	Preterm Birth Caesarean section birth Increase in Maternal morbidity Elevated cortisol Altered immune response Oxidative stress Impaired fetal development & Epigenetic changes	Mental health challenges (e.g., anxiety, depression) Increased care-giver stress Risky coping behaviours Financial hardship Social isolation Fewer prenatal visits Medical insecurity Poor dietary quality	Inadequate sanitation Overcrowding Inadequate nutrition Food insecurity Air pollution
Infancy (0−5 years)	Low birth weight Vitamin deficiencies Brain & peripheral nerve disruptions Motor/cognitive deficits Poor immune development Increase cortisol levels Risk of respiratory & gastrointestinal infection Risk of skin infections	Increase in NICU admissions Longer hospital stays Delayed developmental milestones Poor caregiving quality	Inadequate sanitation Overcrowding Airborne pathogens Environmental hazards Lead poisoning Poor ventilation Unhygienic bedding
Early & Late Childhood (6−12 years)	Neurodevelopmental deficits Verbal cognitive deficits Elevated inflammatory markers Vitamin deficiencies	Mental health challenges (e.g., anxiety, depression, emotional dysregulation) Defiance Hyperactivity Emotional withdrawal Separation anxiety Social exclusion School disruption Unstable peer relationships Child maltreatment Low educational continuity Risky coping behaviours Poor dietary quality	Lack of safe recreational spaces Inadequate sanitation Overcrowding Inadequate nutrition Food insecurity Air pollution
Early & Late Adolescence (13−17 years)	Non-communicable (cardiovascular disease factor, diabetes, high blood pressure) Communicable disease risk (e.g., HIV, Hepatitis C, STD, Tuberculosis)	Mental health challenges (e.g., anxiety, depression, suicidal ideation Educational cessation Maladaptive coping behaviours (e.g., smoking, alcoholism) Stigmatization Poor dietary quality	Inadequate sanitation Overcrowding Inadequate nutrition Air pollution Unsafe neighbourhood Food insecurity
Early & Late Adulthood (18−50 years)	Physiological stress activation (e.g., sympathetic adrenal-medullary (SAM) reactivity, Hypothalamic-pituitary adrenal (HPA) reactivity, Cortisol prolonged expression) Allosteric “wear-and-tear” Non communicable disease established (e.g., cardiovascular disease, disease, respiratory disease, diabetes) Communicable disease risk (e.g., HIV, Hepatitis C, STD)	Mental health challenges (e.g., anxiety, depression) Community violence Financial strain Maladaptive coping behaviours (e.g., substance use, smoking, alcoholism) Social withdrawal Stigmatization Structural discrimination Poor dietary quality	Unstable access to healthcare & disease management Food insecurity Unsafe neighbourhoods Redlining practices
Old Age (55+ years)	Chronic illness (e.g., respiratory disease, cardiovascular disease, Alzheimer’s, neurodegenerative disease) DNA methylation expression Accelerated biological ageing Frailty Balance loss Sensory dysfunction Immune decline Vitamin deficiencies Chronic infectious disease (e.g., Hepatitis C)	Mental health challenges (e.g., depression, anxiety) Social Isolation Ageism Financial strain Medical insecurity Elder abuse Limited social support Structural discrimination	Injuries Poor ventilation Poor insulation Unsafe housing hazards Housing discrimination Air pollution

Notes: The health outcomes presented in Table 1 were not intended to represent an exhaustive list of all outcomes reported in the included studies. Rather, the table reflects a structured synthesis of cross-cutting health outcomes that recur across multiple studies and are theoretically meaningful within a life-course framework. Outcomes were prioritized if they (1) were reported consistently across the literature, (2) reflected key life-course processes such as critical periods, cumulative exposure, or biological embedding, and (3) represented downstream or cross-cutting health consequences plausibly linked to housing insecurity. The selected outcomes are organized by life stage and grouped across biological, psychosocial, and environmental domains to illustrate how housing-related exposures and risk factors accumulate and interact over time, shaping health trajectories and contributing to both short-term outcomes and long-term vulnerabilities across the life course.

## Data Availability

All data for this study are embedded within this manuscript.

## Supplementary Materials

The following supporting information can be downloaded at: https://www.mdpi.com/article/10.3390/ijerph23010091/s1, Figure S1: Pathways linking housing insecurity and health across the life Course.

## Abbreviations

ACE	Adverse Childhood Experiences
COVID-19	Coronavirus disease 2019
DNA	Deoxyribonucleic acid
PRISMA	Preferred Reporting Items for Systematic Reviews and Meta-Analyses
SES	Socio-Economic Status
SDGs	Sustainable Development Goals
UN	United Nations
