# Supplementary figures and images for: Shelter to Survival: Unpacking the Health Impacts of Housing Insecurity Across the Life Course
Source: Int J Environ Res Public Health. 2026 Jan 9;23(1):91. doi: 10.3390/ijerph23010091 (PMC12840798; doi:10.3390/ijerph23010091)

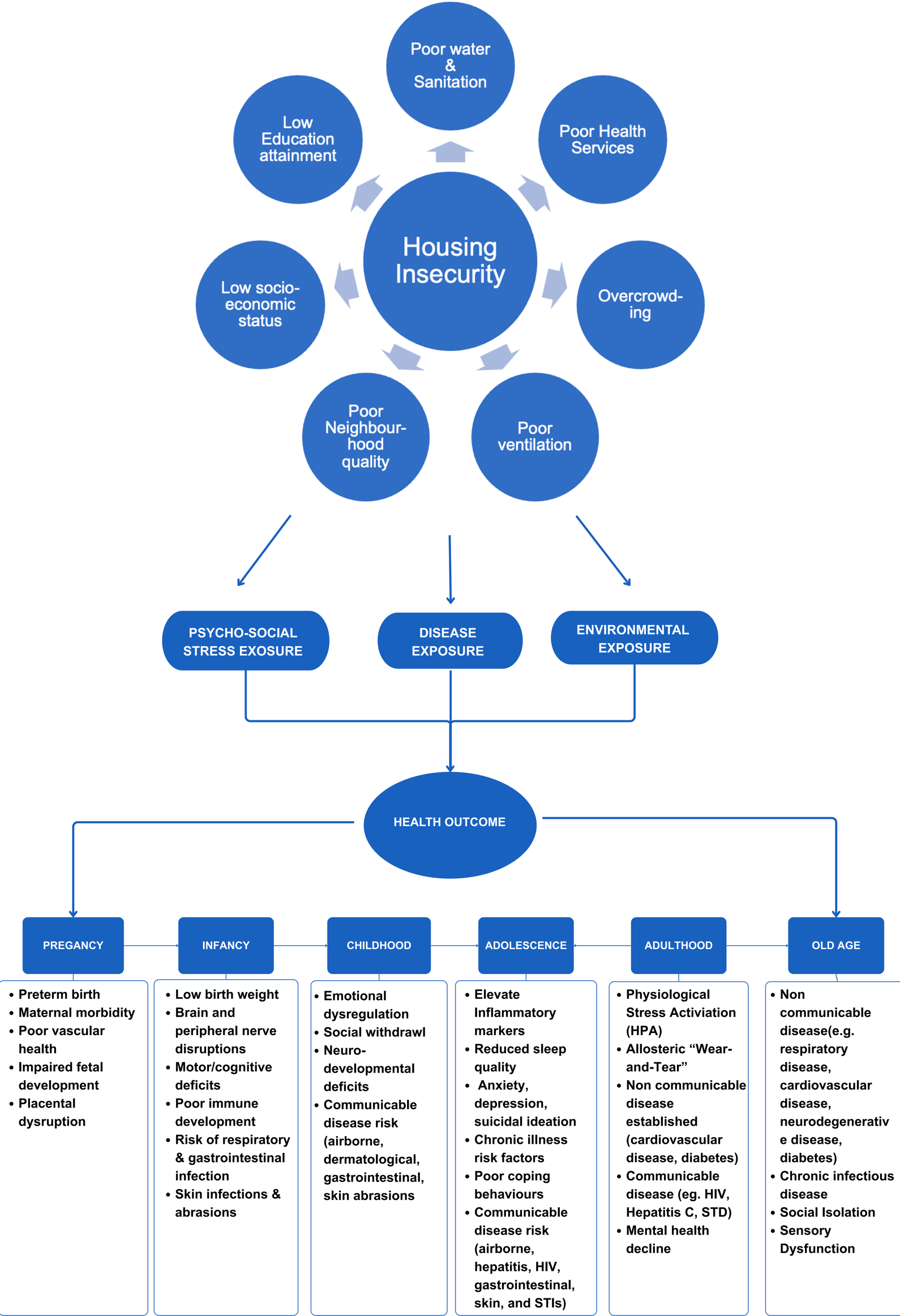

Supplement: Supplementary file 1 [file ijerph-23-00091-s001.zip › ijerph-4028773-supplementary.pdf]
